# Air pollution from wildfires and human health vulnerability in Alaskan communities under climate change

**DOI:** 10.1088/1748-9326/ab9270

**Published:** 2020-08-19

**Authors:** Seung Hyun Lucia Woo, Jia Coco Liu, Xu Yue, Loretta J Mickley, Michelle L Bell

**Affiliations:** 1 School of Forestry and Environmental Studies, Yale University, New Haven, CT, United States of America; 2 Department of Biostatistics, Johns Hopkins Bloomberg School of Public Health, Baltimore, MD, United States of America; 3 Institute of Atmospheric Physics, Chinese Academy of Sciences, Beijing, People’s Republic of China; 4 John A. Paulson School of Engineering and Applied Sciences, Harvard University, Cambridge, MA, United States of America

**Keywords:** air pollution, Alaska, environmental justice, particulate matter, PM_2.5_, smoke, wildfire

## Abstract

Alaskan wildfires are becoming more frequent and severe, but very little is known regarding exposure to wildfire smoke, a risk factor for respiratory and cardiovascular illnesses. We estimated long-term, present-day and future exposure to wildfire-related fine particulate matter (PM_2.5_) across Alaska for the general population and subpopulations to assess vulnerability using observed data for the present day (1997–2010), modelled estimates for the present day (1997–2001), and modelled estimates for the future (2047–2051). First, we assessed wildfire-PM_2.5_ exposure by estimating monthly-average wildfire-specific PM_2.5_ levels across 1997–2010 for 158 Alaskan census tracts, using atmospheric transport modelling based on observed area-burned data. Second, we estimated changes in future (2047–2051) wildfire-PM_2.5_ exposure compared to the present-day (1997–2001) by estimating the monthly-average wildfire-specific PM_2.5_ levels for 29 boroughs/census areas (county-equivalent areas), under the Intergovernmental Panel on Climate Change (IPCC) A1B scenario from an ensemble of 13 climate models. Subpopulation risks for present and future exposure levels were estimated by summing area-weighted exposure levels utilizing the 2000 Census and State of Alaska’s population projections. We assessed vulnerability by several subpopulation characteristics (e.g. race/ethnicity, urbanicity). Wildfire-PM_2.5_ exposure levels during 1997–2010 were highest in interior Alaska during July. Among subpopulations, average summer (June-August) exposure levels for urban dwellers and African-American/Blacks were highest at 9.1 μg m^−3^ and 10 μg m^−3^, respectively. Estimated wildfire-PM_2.5_ varied by Native American tribe, ranging from average summer levels of 2.4 μg m^−3^ to 13 μg m^−3^ for Tlingit-Haida and Alaskan Athabascan tribes, respectively. Estimates indicate that by the mid-21st century, under climate change, almost all of Alaska could be exposed to increases of 100% or more in levels of wildfire-specific PM_2.5_ levels. Exposure to wildfire-PM_2.5_ likely presents a substantial public health burden in the present day for Alaska communities, with different impacts by subpopulation. Under climate change, wildfire smoke could pose an even greater public health risks for most Alaskans.

## 1. Introduction

The smoke resulting from wildfires consists of highly elevated concentrations of fine particulate matter with aerodynamic diameter no larger than 2.5 μm (PM_2.5_), which pose major health risks [1–9]. The chemical structure of particles varies widely and can affect its toxicity [10–12]. Limited but emerging studies demonstrate a consistently significant association between wildfire smoke exposure and risk of respiratory illness, with an increasing size of the population at risk [13–21]. The association is less clear for cardiovascular illness and mortality [13, 20–25]. Susceptible populations to wildfire smoke likely include children, elderly, people with low socio-economic status, and those with chronic conditions such as asthma [18, 26–28], although this is an area for which further research is needed. Few studies have examined different impacts of wildfire smoke by subpopulation [21]; an earlier review recommended studies of diverse populations to help determine sensitive populations [20].

However, the existing literature on the impacts of wildfire events, including exposure and associated health effects, largely focuses on highly-populated, urban communities or the Western U.S [25, 29–36]. In fact, a recent meta-analysis of epidemiological studies of wildfire smoke found no studies of Alaska [37]. Far less is known about many areas of potentially high vulnerability, such as Alaska, where many communities are sparsely populated although wildfire smoke can reach high levels.

A challenge in estimating wildfire smoke exposure in mostly rural Alaska is the limited air quality monitoring network compared to the networks of more urban regions. Accurate attribution of particulate matter exposure specifically to wildfires poses yet another challenge as ambient PM_2.5_ monitors measure total mass of PM_2.5_ from all sources, such as vehicles, road dust, or volcanic ash in addition to wildfire smoke. The few studies that do explore exposure patterns to wildfire smoke in boreal or subarctic communities rely mostly on PM_2.5_ ambient monitor data for indicators of exposure to wildfire smoke exposure, although the monitors do not measure wildfire smoke specifically [2, 6], and do not provide full temporal and spatial coverage. There is a clear need for research that distinguishes between wildfire and non-wildfire PM_2.5_ exposures for rural and urban areas of Alaska across multiple fire seasons.

A small number of studies have examined wildfire smoke under climate change [21]. A recent review identified several studies that reported higher risk from wildfire smoke [38], with limited research on the subsequent exposure and associated health impacts [13, 14, 39, 40]. None of these studies investigated Alaskan populations.

In this study we: (1) estimated population (i.e. non-occupational) exposure to wildfire-specific PM_2.5_ levels for almost the entire state of Alaska during 1997–2010; (2) identified Alaskan subpopulations that are vulnerable to wildfire smoke as those facing high exposures; and (3) projected mid-21st century wildfire smoke exposure under climate change compared to present day levels. For potentially vulnerable populations, we considered several subpopulations, including by urbanicity, race/ethnicity, Native American tribal affiliation, occupation industry, employment and poverty status, income, education, age, and sex.

## 2. Methods

**Figure 1.**
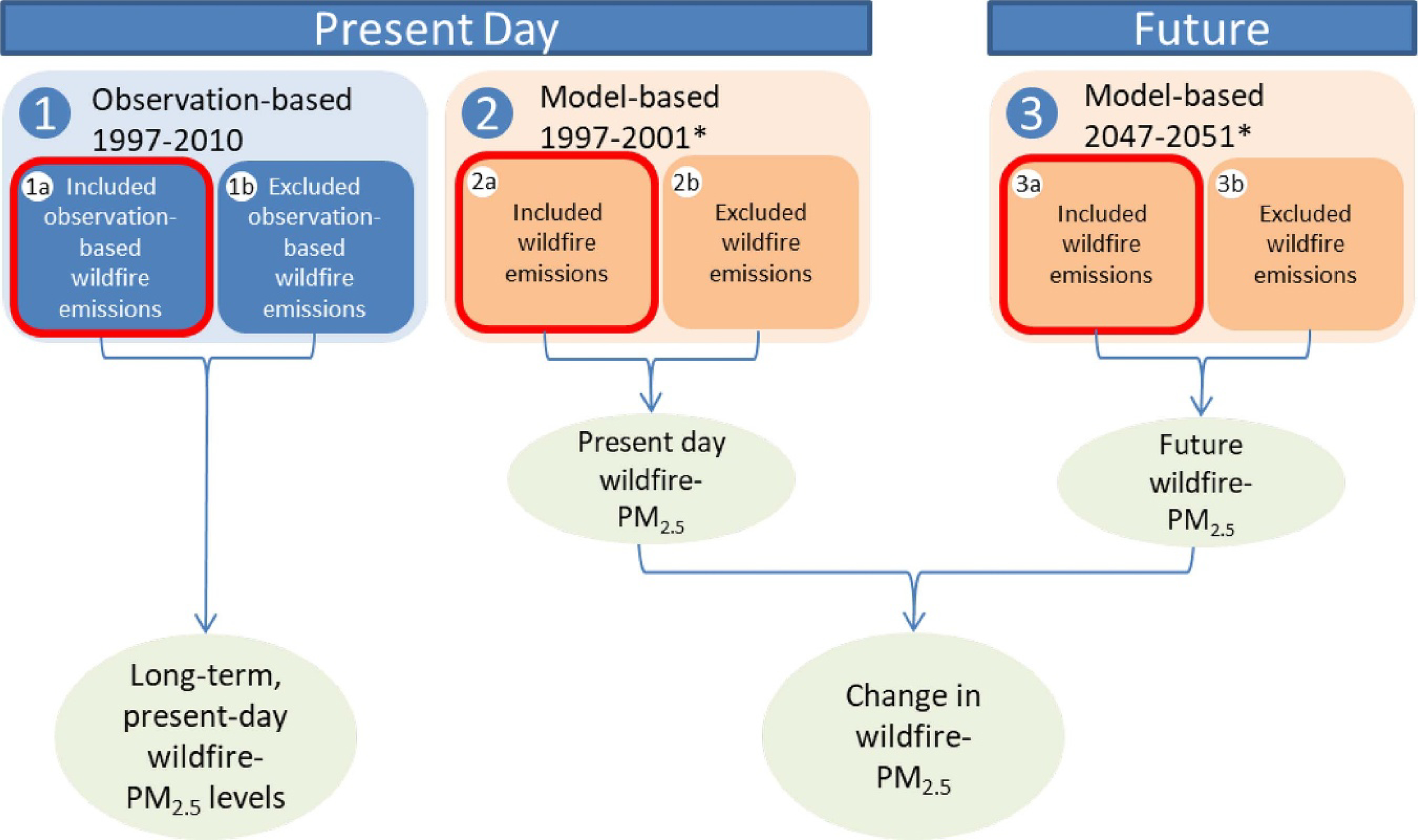
Conceptual schematic of models for analysis.

To overcome limitations of the spatial and temporal coverage of monitors and to estimate exposure specifically from wildfires, we estimated wildfire-specific PM_2.5_ using GEOS-Chem model, a global 3D chemical transport model used extensively to study atmospheric composition relating to air quality [41–43]. For our study, this transport model was used to generate three datasets that approximated monthly average wildfire-specific PM_2.5_ levels across Alaska: 1) observation-based estimates during 1997–2010; 2) present-day modelled estimates during 1997–2001; and 3) future modelled estimates during 2047–2051 under climate change. Figure 1 depicts the modelling system, which is similar to that employed in earlier work [13, 44].

The modelling timeframes are used to represent time periods, not specific days and years. In other words, results for the years 2047–2051 reflect estimated levels for that time period, and the modelling results should not be interpreted for specifically defined days (e.g. 8 July 2049). While we estimate exposure on the basis of a monthly average to help address the uncertainties of climate change modelling, wildfire events do not produce exposures that will be experienced uniformly across the month. Rather, high levels will occur over the span of a few days. Our goal here is not to estimate the number of ‘wildfire events,’ and no consistent definition of a ‘wildfire event’ exists in either the scientific literature or the policy arena. The monthly averages used here should not be interpreted as the average concentrations of pollution *during* a wildfire event, but rather the overall wildfire-related PM_2.5_ averaged over time, including days with and without wildfires. Pollution levels on the actual days of wildfires would be anticipated to be substantially higher.

We utilized the observation-based present-day estimates (depicted as estimates #1 in figure 1) to determine the long-term average population-based (i.e. non-occupational) wildfire-PM_2.5_ levels. The long timeframe of 1997–2010 allowed us to estimate long-term present-day exposures based on observed fire activity. Next, using a fire prediction model that relates meteorology to area burned, we modelled smoke exposure in the present-day and at mid-century (depicted as estimates #2 and #3, respectively, in figure 1). As in Liu *et al* [14], these predictions provide the change in wildfire-PM_2.5_ exposure under climate change over 50 year-intervals (e.g. 2047 estimates compared to 1997 estimates). The model-based estimates (#2 and #3 in figure 1) each use a timeframe of 5 years, with one additional year for spin-up.

For each set of estimates—long-term present-day average (from #1) and the difference between the modelled future and present-day estimates (#3 and #2, representing differences under climate change)—we conducted a vulnerability assessment to determine exposure levels for subpopulations with different characteristics. All analyses were conducted in R v.3.2.2, ArcMap v.10.3.1, and Excel 2013.

We used the GEOS-Chem global 3D Chemical Transport Model (CTM) version 08.03.01 to estimate PM_2.5_ in Alaska specifically due to wildfire emissions. Our modelling system has been applied in previous studies to estimate the wildfire-specific levels of PM_2.5_ for population-level exposure and has been validated on daily and seasonal timescales and with ground-based observations and aircraft-based measurements [45–49]. As an example, in our earlier research, we evaluated estimates from this modelling system against observed area burned and wildfire air pollution [50]. We compared model estimates for organic carbon (OC) to observations from the Interagency Monitoring of Protected Visual Environments (IMPROVE) network, finding high agreement when using interannually varying fire emissions with R^2^ of 0.88 [45]. Detailed evaluations of PM_2.5_ estimates generated by GEOS-Chem, including those for wildfire-specific-PM_2.5_, and descriptions of this modelling system have been published extensively [14, 45–48, 51–59].

The CTM can be driven with assimilated meteorology from the Modern Era Retrospective-analysis for Research and Applications (MERRA) [60] or with the meteorological fields from the freely-running NASA/GISS Model 3 at 4° × 5° latitude by longitude resolution. The assimilated meteorology closely matches observations, while the Model 3 meteorology provides a climatological representation of the present-day and future. Within North America, we used either observation-based wildfire emissions compiled from governmental fire records or the predicted emissions based on observed relationships between area burned and meteorology.

To ensure that the fire prediction model accurately captured Alaskan wildfire activity, we first compiled monthly 1° × 1° area burned for 1980–2010 based on interagency fire reports enmanaged by the National Wildfire Coordinating Group from the Fire and Aviation Management Web Applications (FAMWEB, https://fam.nwcg.gov/fam-web). Each record documented start time, location (longitude and latitude), and area burned for a fire incident. We aggregated approximately 5000 non-duplicated FAMWEB records for Alaska onto 1° × 1° grid cells based on the start months and locations of wildfires [50]. Fuel consumption, which is the amount of biomass burned per unit area, was estimated with 1 km fuel load data from the U.S. Forest Service Fuel Characteristic Classification System and the moisture-dependent burning severity [61]. Total biomass burned, the product of area burned and fuel consumption, was used to estimate emissions of multiple air pollution species by applying specified emission factors [58]. For computational expediency, we than aggregated the monthly 1° × 1° wildfire emissions to 4° × 5° resolution over Alaska.

To derive present-day wildfire-specific PM_2.5_, we next performed two simulations using GEOS-Chem CTM for 1997–2010, with and without the observation-based wildfire emissions (depicted as #1a and #1b, respectively, in figure 1). We applied MERRA meteorology to account for the impacts of transport and deposition on air pollutants. The difference in the simulated surface concentration of PM_2.5_ of these two simulations represents the contribution from wildfire emissions (Supplemental figure 1 (available online at (stacks.iop.org/ERL/00/00000/mmedia))).

To build our fire-prediction model, we aggregated the observed area burned into two ecoregions in Alaska and used stepwise regression to select predictors of area burned, as described in [50]. Potential predictors included meteorological variables (mean and maximum temperature, relative humidity, precipitation, and 500 hPa geopotential height) and fire indices from the Canadian Fire Weather Index system [62] at different seasons and years. We applied the resulting regressions to daily output from 13 climate models in the World Climate Research Programme’s Coupled Model Intercomparison Project phase 3 (CMIP3) [63], yielding area burned for the present-day and future. As in [50] we then calculated fire emissions in the present day and future, using the predicted area burned together with assumptions regarding fuel consumption and emission factors.

Finally, we performed GEOS-Chem simulations for five years in the present-day (1997–2001, with 1996 as spin-up) and future (2047–2051, with 2046 as spin-up), using the emissions derived from the fire prediction model. We also performed two more simulations for the present-day and future in which we excluded the fire emissions. Wildfire-specific pollution is thus defined as the difference between estimated PM_2.5_ values from simulations with and without the fire emissions. All four simulations were driven with meteorology generated by the NASA/GISS Model 3 for the present-day (#2a and #2b, respectively, in figure 1), and the future A1B scenario (#3a and #3b, respectively, in figure 1). We emphasize that this present-day simulation represents a climatological average. The A1B scenario consists of rapid economic growth, peak global population in the mid-century, and balanced energy consumption between fossil-fuel intensive and non-intensive sources [64]. It is comparable to the relatively moderate greenhouse gas concentration trajectory Representative Concentration Pathway (RCP) 4.5.

### 2.1. Long-term present-day, wildfire-PM_*2.5*_ exposure assessment at the Census-tract level

Our present-day wildfire-PM_2.5_ exposure estimates (1997 to 2010) cover almost the entire state of Alaska (latitude from 48°N to 72°N, longitude from 177.5°W to 127.5°W; Supplemental figure 1(a)). When projected to the NAD 1983 Alaska Albers coordinate system, the gridded modelled data with a cell size of 4° latitude × 5° longitude (total of 60 grid cells) overlap with all 158 census tracts in Alaska, except for a portion of one census tract (GEOID #2016000100) in the Aleutians West Census Area. The analysis excludes this portion, which is a fraction of the census tract that contains less than 0.2% of total Alaskan population according to the 2000 Decennial Census. Census tracts are sub-county geographical parcels that remain relatively stable across decades for collecting population data at a fine spatial scale. To estimate the long-term wildfire-PM_2.5_ levels during the present-day, we averaged the estimated monthly wildfire-PM_2.5_ levels in each grid cell from 1997 to 2010. For each census tract, we generated PM_2.5_ wildfire estimates using area-weighted averaging. All individuals residing in a census tract were assigned that tract’s wildfire-specific PM_2.5_ level to estimate exposure.

#### 2.1.1 Vulnerable subpopulation assessment for long-term exposure (1997–2010)

We investigated the vulnerability to wildfire smoke by calculating the average summer (June-August) wildfire-specific PM_2.5_ exposure for 1997–2010 by subpopulations defined by these characteristics: developed settlement type, sex, age, race/ethnicity, Native American tribal affiliation, occupation industry, poverty status, unemployment status, household income, and education level. Except for developed settlement type and Native American tribal affiliation, data for these population characteristics were from the 2000 Decennial Census. Native tribal affiliation data were from the National Historical Geographic Information System [65]. Developed settlement type data were obtained from two sources. We identified 102 census tracts as urban following the 2006 National Center for Health Statistics (NCHS), which classified these tracts as ‘Medium Metro’, ‘Small Metro’, or ‘Micropolitan’ [66]. We also identified 18 census tracts as remote rural (i.e. very limited access to ferry or road), with neither large military presence nor dependence on non-resident commercial fishing according to Goldsmith [67]. The remaining 38 census tracts were identified as rural.

We calculated the summer average wildfire PM_2.5_ exposure across by subpopulation in Alaska as:

$$Y_{i}=\frac{\sum_{j=1}^{N}P_{i,j}x_{j}}{\sum_{j=1}^{N}P_{i,j}}$$

where *Y*_*i*_ is the average summer wildfire-PM_2.5_ exposure level for subpopulation *i* across all census tracts (*N* = 158) in Alaska; *P*_*i,j*_ is the number of persons in subpopulation *i* in census tract *j*; and *x*_*j*_ is the average summer wildfire-PM_2.5_ exposure in census tract *j* (adapted from [14, 68]). Subpopulation types are provided in table 1. We conducted a stratified analysis to investigate whether the summer average exposure levels for various subpopulations differed by the development settlement type of their residence.

### 2.2. Change in wildfire-PM_*2.5*_ exposure in the future under climate change

To estimate the potential future exposure of wildfire smoke, we calculated the change in monthly average wildfire-PM_2.5_ levels for each borough/census area, from 1997–2001 to 2047–2051 under a scenario of climate change. We defined the change in wildfire PM_2.5_ (Δ wildfire-PM_2.5_) levels as the difference between the estimated wildfire-PM_2.5_ for 1997–2001 and 2047–2051. Replicating the area-weighted averaging methodology from the present-day exposure assessment, we then converted the gridded, monthly average wildfire-PM_2.5_ levels for all 29 boroughs and census areas according to the boundaries of 2010. Here we present the wildfire-PM_2.5_ as the percentage change by calculating the ratio of Δ wildfire-PM_2.5_ levels over the present-day (1997–2001) wildfire-PM_2.5_ levels.

#### 2.2.1 Vulnerable subpopulation assessment under climate change

We identified which Alaskan subpopulations may experience higher wildfire smoke exposure under climate change using the same methodology as the present-day vulnerability assessment but with different exposure and population datasets. For exposure we applied the resulting Δ wildfire-PM_2.5_ data under climate change. For population estimates, we leveraged the Alaska Department of Labor and Workforce Development’s population projections to 2042 for each Alaskan borough or census area. This dataset does not reflect population projections under the IPCC A1B scenario, but is based on the 2012 population estimates and historical birth, death, and migration patterns. The projection population data consists of sex and age dimensions in addition to the total population count. Thus, we conducted the vulnerable subpopulation assessment under climate change only for population characteristics of developed settlement type, sex, and age.

To align the 2042 population projection data to the 2047–2051 wildfire PM_2.5_ exposure estimates, we fitted a trend through each borough or census area’s 2012 through 2042 subpopulation projected values (in increments of 5 years) and extrapolated the predicted population value for 2049, the median year among 2047–2051. We then calculated the summer average wildfire-PM_2.5_ levels for all 29 boroughs and census areas as in the present-day assessment.

## 3. Results

### 3.1. Present-day exposure and vulnerability to wildfire PM_*2.5*_

**Figure 2.**
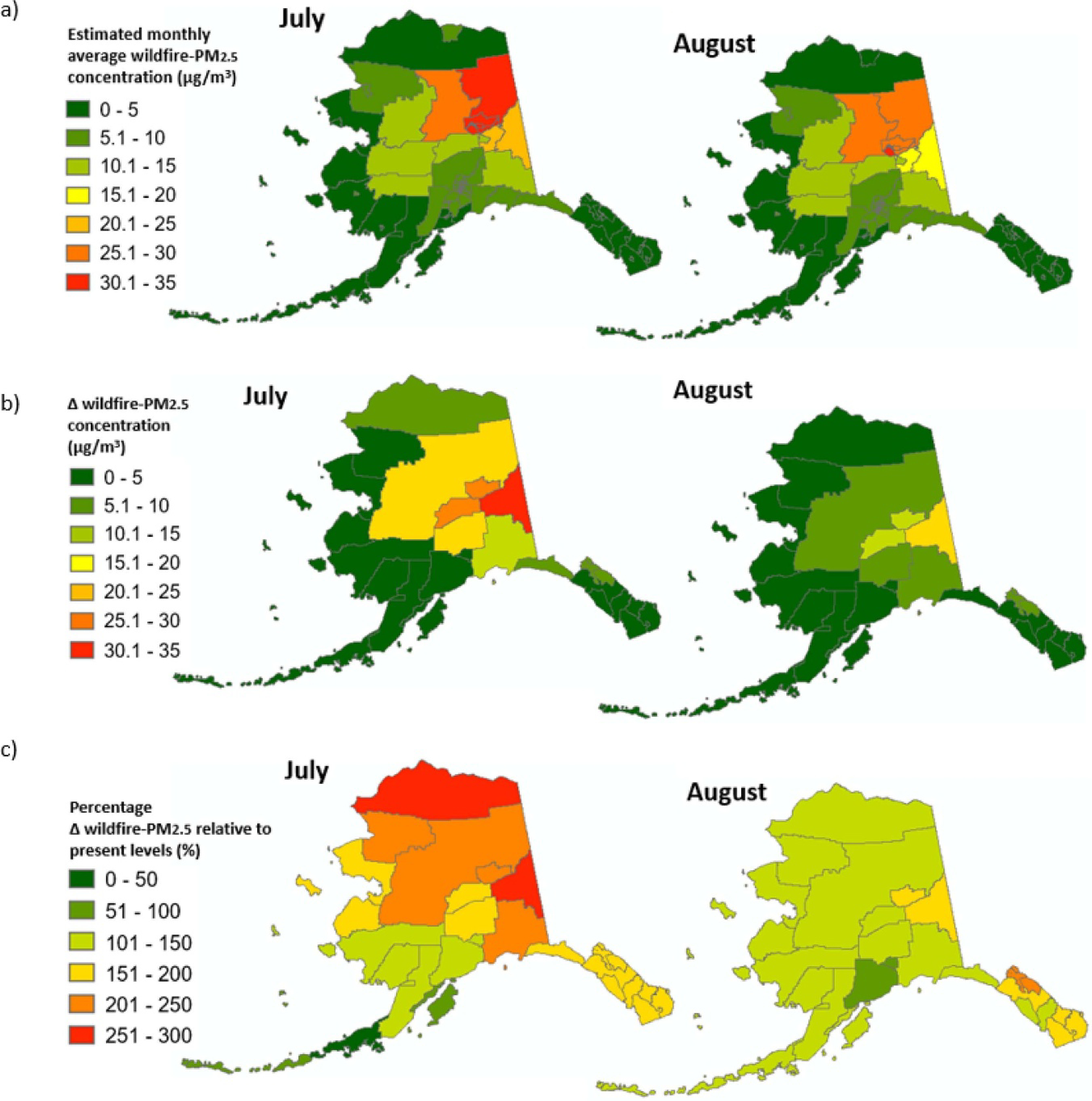
Wildfire-PM_2.5_ levels by borough and census tract in Alaska for July and August. (a) Wildifre-PM_2.5_, present day (1997 to 2010), (b) Change in wildfire-PM_2.5_ for 1997–2001 to 2047–2051 under climate change, (c) Percent change in wildfire-PM_2.5_ exposure relative under climate change (2047–2051) relative to the present-day (1997–2010)

For present day estimates (1997–2010), the monthly average wildfire-PM_2.5_ concentrations at the census tract scale were highest during July and August (figure 2(a)). These exposure estimates represent the PM_2.5_ specifically from wildfires. The long-term exposure levels among the 158 census tracts ranged from 0 to 34 μg m^−3^ during July and 0 to 31 μg m^−3^ during August. The monthly average levels did not exceed 8.5 μg m^−3^ during June and 2.4 μg m^−3^ during non-summer months (i.e. Jan.-May and Sep.-Dec.) in any census tract.

**Figure 3.**
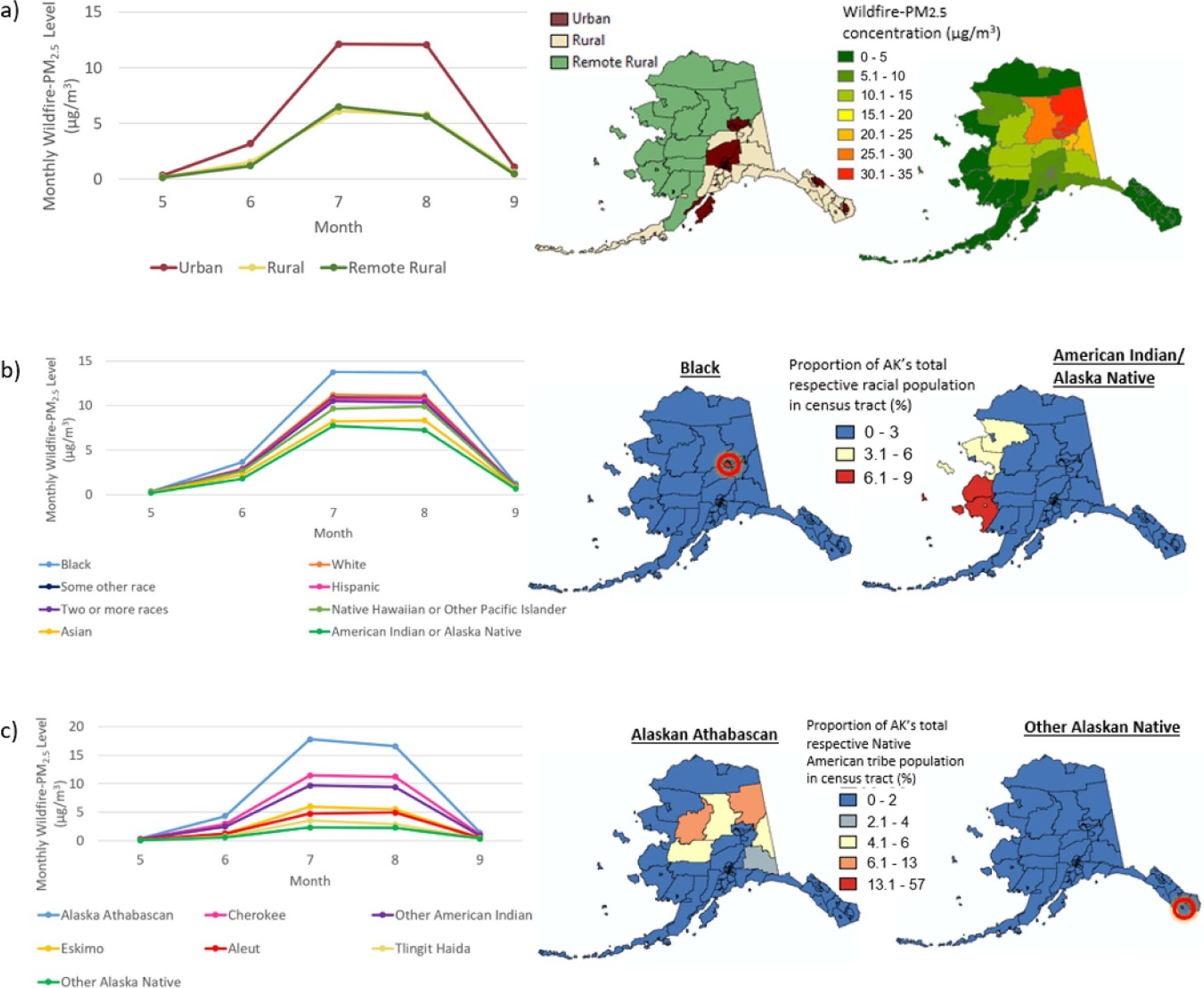
Long-term monthly average wildfire-PM_2.5_ levels during May-Sep. from 1997 to 2010 for Alaskan subpopulations by: (a) developed settlement type; (b) race/ethnicity; and (c) Native American tribal affiliation. Also included are maps of key subpopulations and the long-term present-day (1997–2010) wildfire-PM_2.5_ distribution.

Wildfire-PM_2.5_ exposure levels varied by Alaskan subpopulations, especially for the characteristics of developed environment, race/ethnicity, and Native American tribal affiliation. For developed settlement type, summer (June-Aug.) average wildfire-PM_2.5_ was 9.1 μg m^−3^ for urban area residents and 4.5 μg m^−3^ for rural or remote rural area residents (table 2). Urban exposure levels, which are experienced by about 74% of the Alaskan population, were higher across all summer months, compared to other residents. This differential exposure may reflect the high proportion of the rural and remote rural populations that resided along the Alaskan coastlines, where wildfire smoke exposure was lower than for inland regions (figure 3(a)).

**Figure 4.**
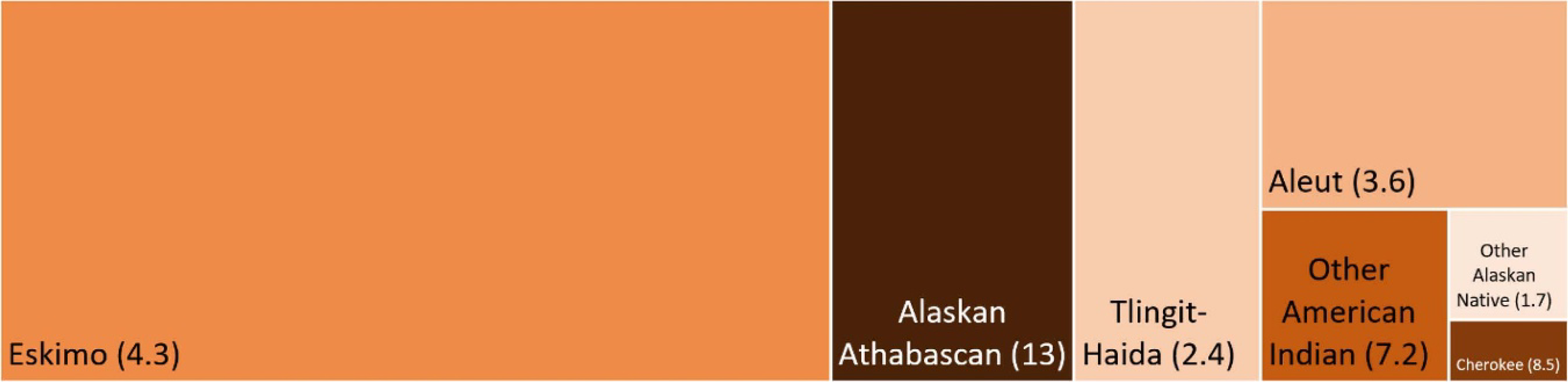
Present day wildfire PM_2.5_ and population size for Native American tribes in Alaska. Each box represents a tribal affiliation. The height of the box represents the level of wildfire PM_2.5_; the width of the box represents the population size; the darkness of the color represents the PM_2.5_ level.

By race and ethnicity, present day summer average wildfire-PM_2.5_ exposure for African-Americans/Blacks was 10 μg m^−3^, which is almost twice as high as that of American Indian and Alaska Native at 5.6 μg m^−3^ (table 2). Although the Native American or Alaska Natives subpopulation had the lowest long-term exposure level overall (5.6 μg m^−3^), we observed variation in wildfire-PM_2.5_ levels by Native American tribal affiliation. Levels were 13 μg m^−3^ and 2.4 μg m^−3^ for Alaskan Athabascan and Tlingit-Haida, respectively (table 2). This difference of 11 μg m^−3^ was the largest disparity in summer average exposure levels among the subpopulation characteristics examined for the vulnerability assessment. Figure 4 depicts the population size and present day wildfire-PM_2.5_ for Native American tribes. Alaska Athabascans, the affiliation with the highest exposure level, has the second largest Native American subpopulation in Alaska with about 12 000 persons in 2000. While the highest exposure was for African-Americans/Blacks, the second highest was for Whites at 8.4 μg m^–3^. which has important public health implications given that this race/ethnicity group has the largest population with 430 000 persons, representing 69% of Alaskans (table 2).

The spatial patterns of wildfire smoke and demographics drive the differences in wildfire-PM_2.5_ exposure (figure 3(b)). For example, about 7% of African-Americans/Blacks residing in Alaska are concentrated in Fairbanks where the long-term exposure level exceeds 30 μg m^−3^ during July and August. In contrast, 6%–9% of the Native American or Alaska Natives reside along the western coast of Alaska where the long-term levels were less than 5 μg m^−3^. Alaska Athabascans mostly lived in the northern interior of Alaska where exposure was highest (figure 3(c)). Upon conducting a stratified analysis by residential developed environment, Alaska Athabascans consistently experienced the highest wildfire-PM_2.5_ exposure levels across all urban, rural, and remote rural environments (table 3).

We did not observe large differences in present day wildfire-PM_2.5_ exposure by occupation industry. Those who worked in the construction industry were found to experience the highest summer average exposure level of 8.2 μg m^−3^, and those who worked in the manufacturing industry were found to experience the lowest exposure level of 5.5 μg m^−3^ (table 4). The Fairbanks area was home to about 2% of the state’s total construction workers, while the Aleutian Islands off the southwestern coast of Alaska was home to 18%–22% of manufacturing workers. For other population characteristics (sex, age, poverty, unemployment, household income level, education level), the differences in long-term wildfire-PM_2.5_ exposure levels among subpopulations did not exceed 1.6 μg m^−3^ between the highest and lowest exposure levels (table 4).

### 3.2. Future exposure and vulnerability to wildfire PM_*2.5*_ under climate change

From 1997–2001 to 2047–2051 under climate change, the wildfire-PM_2.5_ levels in interior Alaska were estimated to increase by at least 15–20 μg m^−3^ in July and 5–10 μg m^−3^ in August (figure 2(b)), representing increases of at least 100% relative to the present-day (figure 2(c)). The changes in wildfire-PM_2.5_ levels under climate change among subpopulations were anticipated to be similar by sex and by age (table 5). However, urban area residents were estimated to experience more than twice the increase in wildfire-PM_2.5_ as remote rural area residents.

## 4. Discussion

This study is one of the first to investigate the wildfire-induced PM_2.5_ exposure patterns and their differences by subpopulations in Alaska, in the present day or under a changing climate. We utilized atmospheric modelling to estimate attribution of fine particulate matter specifically from wildfires, rather than PM_2.5_ total mass from all sources, to fill in spatial and temporal gaps in estimating air pollution from wildfires in high-latitude environments with sparse monitoring networks, which is an advancement over earlier work that bases exposure to wildfires on time and location of events or overall air pollution levels from all sources. A key strength of this study is that we estimated PM_2.5_ specifically from wildfires, as opposed to PM_2.5_ total mass from all sources, and are thereby able to isolate the exposure from this source. Our results on PM_2.5_ exposure suggest that Alaskan communities are at risk for higher health burden from high levels of wildfire smoke exposure in the future under climate change.

Exposure to wildfire smoke, as estimated by wildfire-PM_2.5_, varied across summer months, peaking in July with an additional peak in August, which overlapped with the latter half of the summer when the fuel loads have had time to desiccate from the spring snowmelt [69, 70]. While smoke can travel far from the wildfire source [2, 33], the long-term average exposure levels showed that the Alaskan communities living in the interior, where wildfires are common, experienced higher wildfire-PM_2.5_ exposure relative to coastal communities.

Present day monthly average PM_2.5_ specifically from wildfires were estimated to reach 30–35 μg m^−3^ in 20 census tracts of interior Alaska during July and 12 census tracts during August, which suggests a substantial public health burden in comparison to the U.S. Environmental Protection Agency’s National Ambient Air Quality Standard for PM_2.5_ total mass of 35 μg m^−3^ for the daily standard and 12.0 μg m^−3^ for the annual standard. Importantly, these wildfire-PM_2.5_ exposures are in addition to PM_2.5_ exposure from other sources. While almost all Alaskan communities may experience increased exposure to wildfire-PM_2.5_ in the coming decades under climate change, the interior Alaskan communities, which already have high levels, were estimated to experience the largest increases (figures 2(a)–(c)). Communities in Fairbanks North Star Borough, Southeast Fairbanks Census Area, and Denali Borough may be exposed to an additional 20 μg m^−3^ or higher wildfire-PM_2.5_ (150%–350% increased levels) during July in 2047–2051 relative to 1997–2001. This additional wildfire smoke exposure could translate into a 1.5%–7.5% increase in risk of respiratory hospitalization of those 65 + years for persons residing in interior Alaska due to respiratory causes [14].

These high and prevalent wildfire-PM_2.5_ exposure estimates during the present day and mid-century indicate substantial public health burden of wildfires, which suggest benefits of: 1) public education on protection from wildfire smoke; 2) preparedness on the parts of Alaskan hospitals and healthcare providers for increased influx of patients with illnesses due to wildfire smoke exposure; and 3) consideration in wildfire management plans of differential exposure to wildfire smoke by subpopulations and regions.

The differential wildfire-PM_2.5_ exposure among subpopulations suggests that air quality from wildfires could be an environmental justice issue in Alaska. Some groups may be at a higher risk for health consequences due to their higher exposure to wildfire smoke exposure levels: urban residents compared to rural and remote rural residents; African-Americans/Blacks compared to other races; and Alaska Athabascans compared to other tribal affiliations. This study’s identification of these vulnerable subpopulations highlights the need for decisionmakers—such as Federal and State agencies, Native Tribes and Corporations, and communities—to consider these differential impacts and potentially disproportionate health burdens from exposure to wildfire smoke.

Our research contributes to growing scientific evidence on the health consequences of air pollution from wildfires, with several studies focusing on vulnerability and susceptibility in two ways. Some research focused on identifying those who are most vulnerable due to levels of exposure, as we do here, (e.g. [71–74]). While research on racial/ethnic minorities and socio-economic disadvantaged populations is limited, a small study of 28 low-income homes in Denver, Colorado, U.S. found that indoor air quality in these homes was affected by wildfire pollution [72]. One of the few studies to examine wildfire vulnerability by race considered U.S. Census tracts with a wildfire vulnerability framework that considers both the potential of wildfire and the adaptive capacity [73, 74]. That study found that most persons in the U.S. who live in areas with significant potential for extreme wildfires are white and socio-economically secure, although vulnerability to wildfires was uneven by race and ethnicity with higher vulnerability for Blacks, Hispanics, and Native Americans.

Other research examines whether the health response to exposure to air pollution from wildfires differs for some persons (e.g. [24, 37, 44, 75]). In general, studies found greater health responses for those of lower income, but results were uncertain. A recent review of the health effects of wildfire smoke on respiratory health in North America found evidence that some subpopulations may be more susceptible, but the authors determined that the existing literature was insufficient to draw firm conclusions [37].

Research on the impacts of climate change on forest fires is also growing, with studies finding that forest fires are anticipated to last longer, occur more frequently, and burn hotter, resulting in higher levels of air pollution [44, 50, 76]. Additional research is needed to link climate change and air pollution estimates to environmental disparities frameworks to better understand the potential disproportionate health burden on certain subpopulations from wildfire-related pollution. While we provide one of the first such studies to do so, many research questions remain.

While a strength of our study is the estimation of wildfire-specific PM_2.5_, a limitation is the spatial resolutions of the gridded wildfire-PM_2.5_ estimates. Our grid cell size is 4° latitude by 5° longitude (445 by 153–372 km; figures 2(a)–(b)). More spatially resolved exposure estimates would help distinguish spatial heterogeneity in the concentration field of wildfire PM_2.5_. This could uncover further differences in exposures by subpopulation. Future research with finer spatial resolution for estimates of wildfire-specific air pollution would aid studies of exposures by subpopulation. While the wildfire estimation models have been extensively validated [44, 46], they have not been validated with ground-based air quality monitoring data for 1997 to 2010. When vailable, calibration could help modelled data reflect more real-world exposure levels [14]. EPA did not establish its daily PM_2.5_ monitoring network until 1999, and the monitoring network in Alaska is sparse and clustered in few urban areas. Other metrics of summarizing exposure to wildfire-PM_2.5_, such as the number of days with high or low pollution levels, could be provided in later work, especially as modelling systems advance and as health research provides scientific evidence on what levels are particularly harmful. While further research is needed on the health impacts of wildfire smoke, current research indicates no threshold for the health response for particulate matter from all sources [77, 78]. Other areas of uncertainty include that of the climate modelling, wildfire emissions sources, and details on demographic characteristics. Future work also could examine effect modification by subpopulation characteristic (e.g. how wildfire-PM_2.5_’s association with risk of hospitalization varies by race/ethnicity). While differential exposure does contribute to disparity in health burdens among communities, so does effect modification by population characteristics [79, 80].

## 5. Conclusion

Our findings indicate that interior Alaska currently experiences high levels of wildfire-specific PM_2.5_, and will likely experience significantly increased levels of wildfire-PM_2.5_ exposure and associated health burdens in the future under climate change. Yet, certain subpopulations, including some Alaskan tribes, may experience higher exposure levels and may thus be at higher risk for health complications from wildfire smoke than others. Understanding the distribution of wildfire-PM_2.5_ exposure, and the potential health burden disparities among subpopulations, presents an opportunity for designing effectively targeted public health interventions and informing forest and fire management plans. These concerns are likely to become more relevant in the future as wildfires are anticipated to increase under a changing climate.

## Supplementary Material

Supplemental Material

## Figures and Tables

**Table 1.** Population characteristic definitions and data sources.

Population Characteristic	Characteristic Definition	Data Source

Developed Settlement Type	Persons residing in urban (as defined in 2006 NCHS), rural, or remote rural (as defined in Goldsmith 2007) areas; total count population in each tract	2000 Decennial Census SF1 DP1
Sex	Female or male	2000 Decennial Census SF1 DP1
Age	0–19, 20–64, or ≥65 years	2000 Decennial Census SF1 DP1
Race/Ethnicity	Persons self-identified as single race among White, African-American/Black, American Indian and Alaska Native, Asian, Native Hawaiian and Other Pacific Islanders, and other race, or as two or more races—including as Hispanic	2000 Decennial Census SF1 DP1
Native Tribe Affiliation	Persons who self-identify as American Indian and Alaska Native alone with one tribe only or no tribe specified among Alaska Athabascan, Cherokee, Other American Indian, Eskimo, Aleut, Tlingit-Haida, and Other Alaska Native	2000 Decennial Census SF1a and NHGIS ID: DS146
Occupation Industry	Employed civilian population aged ≥16 years among 13 different industries ranging from agriculture, forestry, fishing and hunting, and mining to public administration	2000 Decennial Census SF3 DP3
Poverty	Persons below the 2000 census poverty thresholds	2000 Decennial Census SF3 QTP34
Unemployment	Persons ≥16 years old in civilian labor force who do not have an officially recorded employment	2000 Decennial Census SF3 DP3
Household Income	Household income levels in 1999	2000 Decennial Census SF3 DP3
Education Level	Persons ≥25 years old who had highest educational attainment of less than high school, high school, some college or associate’s, bachelor’s, or graduate school	2000 Decennial Census SF3 DP2

**Table 2.** Summer average wildfire-PM_2.5_ present day levels and subpopulation size by population characteristic.

Population Characteristic	Summer (June-August) Average Exposure Level	Year 2000 Population Size (% of Total Population)

*Developed Settlement Type*		
Urban	9.1 μg m^−3^	461 139 (74%)
Rural	4.5 μg m^−3^	105 674 (17%)
Remote Rural	4.5 μg m^−3^	60 119 (10%)
*Race/Ethnicity*		
African-American/Black	10 μg m^−3^	22 000 (3%)
White	8.4 μg m^−3^	43 0000 (69%)
Some Other Race	8.2 μg m^−3^	10 000 (2%)
Hispanic	8.1 μg m^−3^	26 000 (4%)
Two or More Races	7.9 μg m^−3^	34 000 (5%)
Native Hawaiian & Other Pacific Islander	7.4 μg m^−3^	3300 (1%)
Asian	6.3 μg m^−3^	25 000 (4%)
American Indian & Alaska Native	5.6 μg m^−3^	98 000 (16%)
Native American Tribal Affiliation Alaskan Athabascan	13 μg m^−3^	12 000 (15%)
Cherokee	8.5 μg m^−3^	960 (1%)
Other American Indian	7.2 μg m^−3^	4200 (5%)
Eskimo	4.3 μg m^−3^	41 000 (53%)
Aleut	3.6 μg m^−3^	8300 (11%)
Tlingit-Haida	2.4 μg m^−3^	9200 (12%)
Other Alaskan Native	1.7 μg m^−3^	1700 (2%)

**Table 3.** Present day summer average wildfire-PM_2.5_ exposure levels and population sizes by Native American tribal affiliation by development type: urban, rural, and remote rural environment.

*Native American* *Tribal Affiliation*	Urban		Rural		Remote Rural	
	*Summer Average Exposure Level [μg m^−3^]*	*Population Size (% of Total Population)*	*Summer Average Exposure Level [μg m^−3^]*	*Population Size (% of Total Population)*	*Summer Average Exposure Level [μg m^−3^]*	*Population Size (% of Total Population)*

Alaska Athabascan	15	5283 (20%)	8.8	2255 (18%)	13	4372 (11%)
Cherokee	9.7	758 (3%)	4.5	164 (1%)	4.0	40. (<1%)
Other American Indian	8.9	2, 655 (10%)	4.2	771 (6%)	4.0	725 (2%)
Eskimo	8.9	7760 (30%)	3.9	1014 (8%)	3.2	32707 (84%)
Aleut	5.2	4315 (16%)	1.8	3098 (24%)	2.4	869 (2%)
Tlingit-Haida	3.0	4908 (19%)	1.6	4207 (33%)	4.3	40. (<1%)
Other Alaska Native	3.7	528 (2%)	0.8	1139 (9%)	3.7	30. (<1%)

**Table 4.** Summer average wildfire-PM_2.5_ exposure levels (1997–2010) and subpopulation size, by population characteristic.

Population Characteristic	Summer (June to August) Average Wildfire-PM_2.5_	Year 2000 Population Size (% of Total Population)
*Sex*		
Male	7.9 μg m^−3^	324 112 (52%)
Female	7.9 μg m^−3^	302 820 (48%)
*Age*		
<20 years	7.9 μg m^−3^	208 117 (33%)
20 to 64 years	7.9 μg m^−3^	383 116 (61%)
≥65 years	7.4 μg m^−3^	35 699 (6%)
*Poverty Status*		
Above Poverty	7.9 μg m^−3^	555 359 (91%)
Below Poverty	7.5 μg m^−3^	57 602 (9%)
*Unemployment Status (only civilian labor force)*		
Employed	7.7 μg m^−3^	281 532 (91%)
Unemployed	7.6 μg m^−3^	27 953 (9%)
*Household Income Level (annual)*		
<$15 000	8.2 μg m^−3^	23 453 (11%)
$15 000–$34 999	8.1 μg m^−3^	47 942 (22%)
$35 000–$49 999	8.1 μg m^−3^	35 519 (16%)
$50 000–$99 999	7.9 μg m^−3^	79 283 (36%)
>$99 999	7.7 μg m^−3^	35 607 (16%)
*Education Level (≥25 years)*		
Less than High School	6.9 μg m^−3^	44 282 (12%)
High School	7.5 μg m^−3^	105 812 (28%)
Some College or Associate’s	8.1 μg m^−3^	135 655 (36%)
Bachelor’s	7.9 μg m^−3^	61 196 (16%)
Graduate School	8.5 μg m^−3^	32 611 (9%)
*Occupation Industry, Employed Civilian (≥16 years)*		
Construction	8.2 μg m^−3^	20 534 (7%)
Educational, Health & Social Services	8.1 μg m^−3^	61 165 (22%)
Retail Trade	8.0 μg m^−3^	32 638 (12%)
Other Services (except Public Administration)	8.0 μg m^−3^	15 866 (6%)
Arts, Entertainment, Recreation, Accommodation & Food Services	8.0 μg m^−3^	24 099 (9%)
Professional, Scientific, Management, Administrative, & Waste Management Services	7.9 μg m^−3^	21 322 (8%)
Wholesale Trade	7.9 μg m^−3^	7215 (3%)
Transportation & Warehousing, and Utilities	7.8 μg m^−3^	25 043 (9%)
Information	7.8 μg m^−3^	7652 (3%)
Finance, Insurance, Real Estate, and Rental and Leasing	7.8 μg m^−3^	12 934 (5%)
Pubic Administration	7.3 μg m^−3^	30 070 (11%)
Agriculture, Forestry, Fishing and Hunting, and Mining	6.0 μg m^−3^	14 000 (5%)
Manufacturing	5.5 μg m^−3^	9200 (3%)

**Table 5.** Change in summer average wildfire-PM_2.5_ exposure levels from 1997–2001 to 2047–2051 under climate change by key population characteristics, including subpopulation sizes.

Population Characteristic	Change in Summer (June—August) Average Wildfire-PM_2.5_ Level	Approximate Year 2049 Population Size (% of Total)

*Sex*		
Male	+3.4 μg m^−3^	487 071 (51%)
Female	+3.4 μg m^−3^	474 113 (49%)
*Age*		
<20 years	+3.4 μg m^−3^	271 483 (28%)
20 to 64 years	+3.3 μg m^−3^	579 575 (60%)
≥65 years	+3.3 μg m^−3^	120 907 (12%)
*Developed Settlement Type*		
Urban	+3.8 μg m^−3^	756 108 (79%)
Rural	+2.4 μg m^−3^	120 549 (13%)
Remote Rural	+1.5 μg m^−3^	84 721 (9%)
