# Supplementary material for: Air pollution from wildfires and human health vulnerability in Alaskan communities under climate change
Source: Environ Res Lett. Author manuscript; Available in PMC 2021 Aug 18. (PMC8372693; doi:10.1088/1748-9326/ab9270)
Supplement: Supplemental Material [file NIHMS1703332-supplement-Supplemental_Material.pdf]

Supplemental Material: Air pollution from wildfires and human health vulnerability in Alaskan communities under climate change

Seung Hyun Lucia Woo, Jia Coco Liu, Xu Yue, Loretta J. Mickley, and Michelle L. Bell

Supplemental Figure 1: Grid for modeled wildfire-$PM_{2.5}$ estimates for Alaska*:*

a) FAMWEB-based wildfire-$PM_{2.5}$ grid for 1997-2010 estimates, with 158 Alaskan census tract boundaries for year 2000

b) Change in wildfire-$PM_{2.5}$ (2047-2051 minus 1997-2001) grid, with 29 Alaskan borough/census area (county-equivalent area) boundaries for year 2010

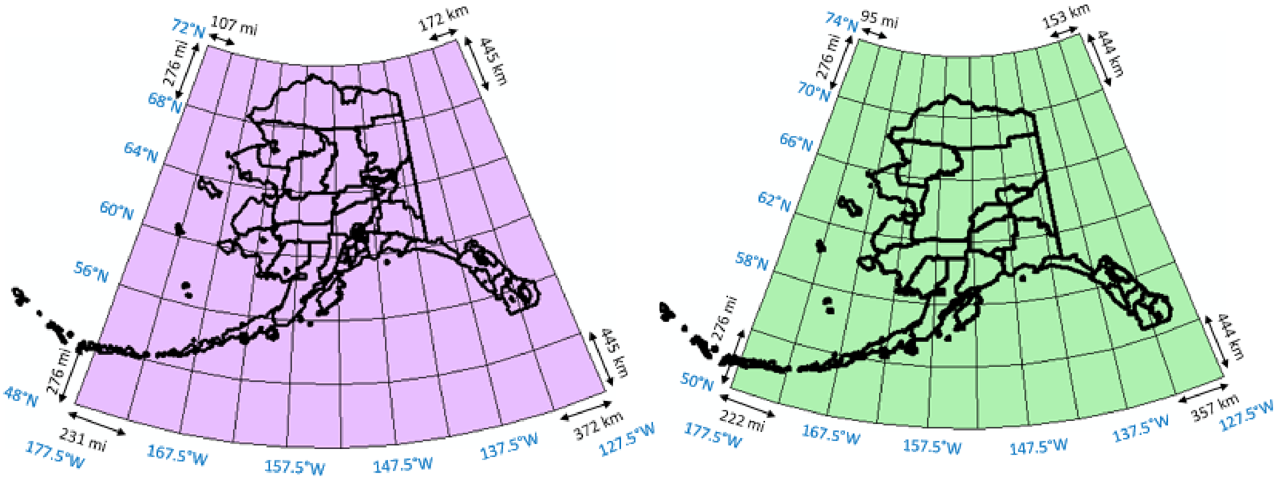

(a) (b)
